# Subcutaneous adipose tissue transplantation in diet-induced obese mice attenuates metabolic dysregulation while removal exacerbates it

**DOI:** 10.1002/phy2.15

**Published:** 2013-07-08

**Authors:** Michelle T Foster, Samir Softic, Jody Caldwell, Rohit Kohli, Annette D deKloet, Randy J Seeley

**Affiliations:** 1 Department of Psychiatry, Obesity Research Center, University of CincinnatiCincinnati, Ohio; 2 Division of Gastroenterology, Hepatology, and Nutrition, Department of Pediatrics, Cincinnati Children's Hospital Medical CenterCincinnati, Ohio; 3 Department of Pathology and Molecular Medicine, Obesity Research Center, University of CincinnatiCincinnati, Ohio; 4 Department of Internal Medicine, Obesity Research Center, University of CincinnatiCincinnati, Ohio

**Keywords:** Glucose intolerance, insulin resistance, lipectomy, peripheral adipose tissue, type-2-diabetes, visceral obesity

## Abstract

Adipose tissue distribution is an important determinant of obesity-related comorbidities. It is well established that central obesity (visceral adipose tissue accumulation) is a risk factor for many adverse health consequences such as dyslipidemia, insulin resistance, and type-2-diabetes. We hypothesize that the metabolic dysregulation that occurs following high fat diet-induced increases in adiposity are due to alterations in visceral adipose tissue function which influence lipid flux to the liver via the portal vein. This metabolic pathology is not exclusively due to increases in visceral adipose tissue mass but also driven by intrinsic characteristics of this particular depot. In Experiment 1, high fat diet (HFD)-induced obese control (abdominal incision, but no fat manipulation) or autologous (excision and subsequent relocation of adipose tissue) subcutaneous tissue transplantation to the visceral cavity. In Experiment 2, mice received control surgery, subcutaneous fat removal, or heterotransplantation (tissue from obese donor) to the visceral cavity. Body composition analysis and glucose tolerance tests were performed 4 weeks postsurgery. Adipose mass and portal adipokines, cytokines, lipids, and insulin were measured from samples collected at 5 weeks postsurgery. Auto- and heterotransplantation in obese mice improved glucose tolerance, decreased systemic insulin concentration, and reduced portal lipids and hepatic triglycerides compared with HFD controls. Heterotransplantation of subcutaneous adipose tissue to the visceral cavity in obese mice restored hepatic insulin sensitivity and reduced insulin and leptin concentrations to chow control levels. Fat removal, however, as an independent procedure exacerbated obesity-induced increases in leptin and insulin concentrations. Overall subcutaneous adipose tissue protects against aspects of metabolic dysregulation in obese mice. Transplantation-induced improvements do not occur via enhanced storage of lipid in adipose tissue, however, altered hepatic lipid regulation may play a contributory role.

## Introduction

Adipose tissue distribution plays a fundamental role in the prevalence of obesity-related comorbidities. In humans, central obesity, characterized by accumulation of visceral adipose tissue, has a greater association with metabolic impairments such as dyslipidemia (Kissebah et al. 1976), hypertension (Cassano et al. 1990; Seidell et al. 1991), insulin resistance, and type-2-diabetes (Chan et al. 1994; Carey et al. 1997; Astrup et al. 2007) than increases in subcutaneous adipose tissue stores (Seidell et al. 1990; Cefalu et al. 1995; Snijder et al. 2004; Kuk et al. 2006). Although the specific mechanisms for metabolic differences are not known, pathophysiological consequences of visceral adipose tissue accumulation are commonly attributed to adipose mass, location, and/or adipocyte-specific physiology.

Adipose tissue manipulation, removal, or transplantation studies delineate whether the metabolic risks of excess visceral adipose tissue are due to the anatomical location or due to the intrinsic properties of adipose depots. Removal of visceral adipose tissue in overweight rodents (Foster et al. 2010) and humans (Thorne et al. 2002) has been demonstrated to reverse adverse metabolic effects such as insulin resistance and glucose intolerance; this, however, is controversial in humans (Thorne et al. 2002; Fabbrini et al. 2010). In humans data predicts that increased visceral fat mass is a fundamental problem in obesity-related metabolic disorders; however, data from rodent studies support an alternative view. In rodents, increasing visceral/intraabdominal adipose tissue mass via heterologous (adipose tissue from a donor) transplantation, unexpectedly improved glucose tolerance and insulin sensitivity (Hocking et al. 2008; Tran et al. 2008; Foster et al. 2010). The degree of improvement, however, appears to depend upon the physiological properties of the adipose tissue transplanted. For example, subcutaneous (Konrad et al. 2007; Hocking et al. 2008; Tran et al. 2008; Foster et al. 2011b), but not visceral (Tran et al. 2008; Foster et al. 2011a) adipose tissue transplantation improved insulin sensitivity and glucose tolerance. Overall, these studies demonstrate that (1) increased intraabdominal/visceral adipose tissue mass alone is not sufficient to induce metabolic dysregulation, hence transplantation of healthy adipose tissue does not mimic diet-induced visceral adipose tissue accumulation, (2) transplantation-induced metabolic outcomes depend on the intrinsic characteristics of the relocated adipose depot, and (3) detrimental effects of visceral adipose accumulation are due to both proximal location to the portal vein and depot characteristics. Depot/adipocyte-specific variations include, but are not limited to, differences in lipid metabolism, lipolytic capacity, insulin action, and adipokine expression (Pujol et al. 2003; Giorgino et al. 2005; Romero Mdel et al. 2009; Palou et al. 2010). The inherent characteristics of adipose depots that drive transplantation-induced differences, however, are not known.

Additional consequences specific to visceral located subcutaneous transplants include decreased adipocyte cell size, adiposity, and body mass (Hocking et al. 2008; Tran et al. 2008; Foster et al. 2011b). We have observed similar outcomes with autotransplantation (adipose depot removal with subsequent transplantation to another location within the same animal) (Foster et al. 2011b). Unlike heterologous transplantation, autologous transplantation is a procedure that enables the assessment of physiological alterations that occur as a result of both fat removal and transplantation without initially altering total adiposity. The mechanisms responsible for subcutaneous transplant-induced decreases in adiposity are not known and have not been demonstrated in an obese model. Therefore, Experiment 1 evaluated adiposity and alterations in portal lipids following the relocation of subcutaneous adipose tissue to the visceral cavity in diet-induced obese mice.

Previous studies in rodents have demonstrated improved glucose tolerance, reversal of insulin resistance, and reduced adipo/cytokine levels following the removal of visceral or intraabdominal adipose tissue (Barzilai et al. 1999; Gabriely et al. 2002; Pitombo et al. 2006). Few, however, have investigated the direct metabolic effects of subcutaneous adipose tissue removal. To our knowledge only two rodent studies have investigated the effects of subcutaneous adipose tissue removal on metabolic indices in diet-induced obese mice. While in chow or high fat diet (HFD)-fed mice the removal of subcutaneous adipose tissue had no effect on glucose regulation (Shi et al. 2007), subcutaneous adipose tissue removal in hamsters resulted in hypertriglyceridemia and increased liver lipid deposition (Weber et al. 2000). Therefore, Experiment 2 compared the relative contribution of the individual components of autologous transplantation, adipose removal contrasted with transplantation.

## Research Design and Methods

### Animals

Adult male C57BL/6 mice (Jackson Laboratory, Bar Harbor, Maine) (∼20 g) were group housed in Experiment 1 and individually housed in Experiment 2 under controlled conditions (12:12 light–dark cycle, 50–60% humidity, and 25°C). A week following arrival, during acclimation, mice had free access to low-fat pelleted chow (Harlan Teklad LM485, Madison, WI; 3.1 kcal/g) and water. Subsequently, unless otherwise noted to remain on standard chow, mice had free access to a high-fat butter oil-based diet (Research Diets, New Brunswick, NJ; D12451; 45% fat; 4.73 kcal/g) for 6 weeks prior to surgery and then postsurgery to termination. In Experiment 1, mice were moved to individual housing conditions 1 week prior to experiment start. Body mass and food intake were recorded weekly postsurgery. Procedures were approved by the University of Cincinnati Institutional Animal Care and Use Committee.

### Fat removal and transplantation surgeries

**Figure 1:**
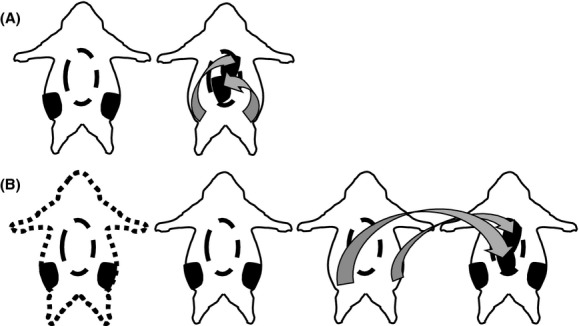
Experimental groups: solid animal outline represents high fat diet (HFD) fed, while square dot is chow fed. Oval dash in the center is the visceral cavity. Black bilateral structures are the inguinal adipose depots. Arrows indicate where adipose tissue was removed and relocated to. (A) Experiment 1 – HFD control and HFD inguinal autotransplantation. (B) Experiment 2 – Chow control, HFD control, HFD bilateral inguinal removal, and HFD inguinal heterotransplantation.

Figure 1 picture represent Experiments 1 and 2 groups. Following anesthetization with isoflurane surgeries were performed through a midventral abdominal incision. In Experiment 1, mice with adipose tissue relocation (excision of fat from one site with subsequent relocation within the same mouse) were termed autotransplantation (W/in). For the W/in group subcutaneous adipose tissue removal included bilateral excision of inguinal white adipose tissue (IWAT) where the skin of the dorsal hind limb was separated from the musculature and a total of ∼300 mg of fat was removed. The excised subcutaneous adipose tissue was immediately transplanted to the visceral cavity. The transplants were attached, via VETBOND tissue adhesive (3M Center, St. Paul, MN), anterior to the inferior gastric curvature (∼150 mg) in close proximity to the hepatic vasculature and to mesenteric fat (∼150 mg) near splanchnic circulation. In respective controls (controls: mice with abdominal incisions, but no fat manipulation) VETBOND was applied to adipose tissue attached to the stomach and cecum.

In Experiment 2, obese mice with inguinal adipose tissue excision were termed lipectomy (Lipx), whereas mice that received these donor transplants from lipectomized siblings were termed heterotransplantation (Trans Sub). Control surgeries of chow (Chow Control) and HFD (HFD Control)-fed mice and the independent procedures of adipose tissue excision or transplantation occurred as previously described for W/in mice. Amount of tissue removed and transplanted to donor was ∼300 g.

For both experiments, after muscle and skin were closed with absorbable suture meloxicam analgesic (0.025 mg/10 g body weight) was injected subcutaneously.

### Glucose tolerance test

Intraperitoneal glucose tolerance tests (ipGTTs) in Experiments 1 and 2 were conducted presurgery and 4 weeks postsurgery. Following an overnight fast (16-h), baseline blood glucose was obtained from the tail vein and in Experiment 1, only ∼250 μL of blood was collected to measure plasma insulin concentration. Mice then received a 1.5 g/kg dextrose injection and blood glucose was assessed from tail vein blood samples 15, 30, 45, 60, and 120 min postinjection.

### Body composition analysis

In Experiment 2, body composition was analyzed via quantitative nuclear magnetic resonance (NMR; Echo MRI Whole Body Composition Analyzer; Echo Medical Systems, Houston, TX) 2 days before experiment termination.

### Terminal procedures (blood collection and tissue harvesting)

For Experiment 1, 5 weeks postsurgery ad libtum fed mice were weighed then anesthetized with isoflurane. Blood was collected from the hepatic portal vein (∼150 μL) and a portion of the right lobe of the liver was snap frozen in liquid nitrogen, plasma and liver were stored at −80°C. Inguinal (IWAT), epididymal (EWAT), perirenal (PWAT), and visceral white adipose tissue (VWAT) were dissected and weighed (bilateral depots were summed). The inguinal depot of transplanted and control mice was collected and placed in formalin for histology. In Experiment 2, 5 weeks postsurgery mice were fasted for 4 h and injected ip with saline or insulin (1 mU/g; SAFC Biosciences, Inc., Lenexa, KS) ∼15 min before being anesthetized with isoflurane. Blood and tissue collection was performed as described Experiment 1.

### Quantitative lipid assays

In Experiments 1 and 2, liver samples (50 mg) were homogenized in 50 mmol/L Tris·HCl buffer, pH 7.4, containing 150 mmol/L NaCl, 1 mmol/L ethylenediaminetetra acetic acid, and 1 μmol/L phenylmethylsulfonyl fluoride. Samples were centrifuged and lipid supernatant was immediately used to determine triacylglycerol concentration. Commercially available kits were used to measure plasma and liver triacylglycerol (Randox Laboratories, Crumlin, U.K.) along with plasma cholesterol (Thermo Fisher Scientific, Middletown, VA) and nonesterified fatty acids (Wako, Richmond, VA) per the manufacturers’ instructions. Absorbance was measured using a microplate reader (Synergy HT; BioTek Instruments, Richmond, VA).

### Adipose tissue histology

In Experiments 1 and 2, WAT histology was performed according to the method of Foster and Bartness (2006). Each inguinal depot was sliced across its extent at 10 μm using a rotary microtome (American Optical Instrument, Buffalo, NY). Slides were grouped into levels of approximately <100 μm, and five slides with three slice sections from each sample were analyzed by light microscopy for average relative cell size (pixels) using ImageJ (NIH, Bethesda, MD).

### Western blot (hepatic insulin sensitivity)

In Experiment 2, liver samples were homogenized and protein was extracted. Total Akt (Primary-1:1000; Cell Signaling, Danvers, MA) and phospho-Akt (pAKT; 1:1000; Cell Signaling, Danvers, MA) protein production was assessed via western blot analysis. Specifically chemiluminescence-induced (GE Healthcare, Chalfont St32 Giles, U.K.) densitometry readings were visualized on film. Density was quantified using Image J. Background was subtracted from each sample, and pAKT samples were normalized to total AKT and expressed as percent of respective saline-injected controls.

### Mouse plasma adipokine assay

In Experiment 2, portal plasma insulin, leptin, resistin, adiponectin, monocyte chemotactic protein-1(MCP-1), interleukin 6 (IL-6), and plasminogen activator inhibitor-1 (PAI-1) concentrations were determined using commercial kits (EMD Millipore Corporation, Billerica, MA). All samples were processed according to the manufacturers’ protocols and analyzed on a Luminex instrument (LX200; Millipore, Austin, TX).

### Statistical analysis

Data are expressed as mean ± standard error of the mean (SEM). Experiments 1 and 2 were ran independently as are the statistics. Within the independent experiments comparisons among multiple groups were performed using one-way between-subjects analysis of variance (ANOVA) (SPSS for Windows, release 11.5.0; SPSS, Chicago, IL). This was performed on most dependent variables including body weight, NMR body composition, tissue mass, area under the curve (AUC), adipose tissue cell size, lipid measurements, obesity markers, and pAKT. ipGTT data were analyzed using two-way ANOVA with group and time as the factors (Experiment 1: 2 × 6, Experiment 2: 4 × 6). Post hoc tests of individual groups were made using Tukey's tests. For all experiments, differences among groups were considered statistically significant if *P* ≤ 0.05. Exact probabilities and test values were omitted for simplicity and clarity of presentation of the results.

## Results

### Experiment 1 – Lipid and adiposity alterations following subcutaneous autotransplantation in obese mice

#### Glucose tolerance test

**Figure 2:**
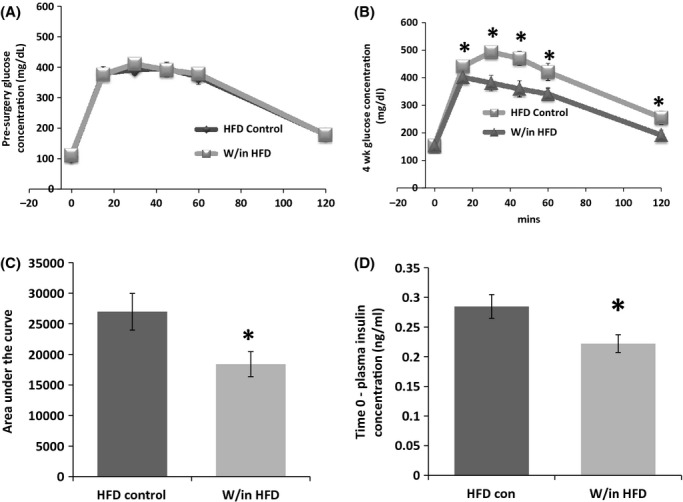
Experiment 1: glucose concentration (mg/dL) at 0, 12, 30, 45, 60, and 120 min after glucose injection before surgery (A) and 4 weeks postsurgery (B). Four weeks postautotransplantation glucose was decreased 15, 30, 45, and 60 min postinjection, hence area under the curve (AUC) (C) was as well compared with control (*P ≤ 0.05). Insulin concentration was decreased following an overnight fast (*P ≤ 0.05 vs. control) (D).

Groups (HFD control *n* = 7 and W/in HFD *n* = 11) were matched for their average body mass and the curve of the presurgery GTT (Fig. 2A). Subcutaneous autotransplantation in HFD mice significantly decreased blood glucose at 15, 30, 45, 60, and 120 min compared with HFD controls (*P* ≤ 0.05, Fig. 2B); consequently AUC was significantly decreased (*P* ≤ 0.05, Fig. 2C). In addition, plasma insulin concentration, from blood collected after the 16 h fast but before glucose injection, was significantly decreased in HFD mice with subcutaneous autotransplantation (*P* ≤ 0.05, Fig. 2D).

#### Food intake, body weight, and adipose tissue mass

Cumulative food intake did not differ among groups (HFD Control 405.14 ± 5.02 kcals and W/in HFD 404.2 ± 3.03 kcals). At termination, 5 weeks postsurgery, subcutaneous adipose tissue autotransplantation did not alter body weight in HFD mice (Table 1). PWAT and VWAT were not different among the groups. EWAT, however, was significantly decreased following autotransplantation compared with controls (*P* ≤ 0.05). IWAT mass was significantly decreased in autotransplantated mice because it was the excised depot (*P* ≤ 0.05).

**Table 1:** Experiment 1: body mass (g) and absolute individual dissected adipose tissue mass (bilateral mass – g)

	Body weight (g)	VWAT (g)	EWAT (g)	IWAT (g)	PWAT (g)
HFD control	31.58 ± 1.35	0.47 ± 0.04	1.30 ± 0.16*	0.38 ± 0.06*	0.52 ± 0.06
W/in HFD	30.06 ± 0.64	0.51 ± 0.05	0.90 ± 0.08	0.11 ± 0.01	0.40 ± 0.04

Values are means ± SEM. VWAT, visceral white adipose tissue; EWAT, epididymal white adipose tissue; IWAT, inguinal white adipose tissue; PWAT, perirenal white adipose tissue; HFD, high-fat diet.

* *P* ≤ 0.05 control versus surgery.

#### Adipose tissue histology and lipid profile

**Figure 3:**
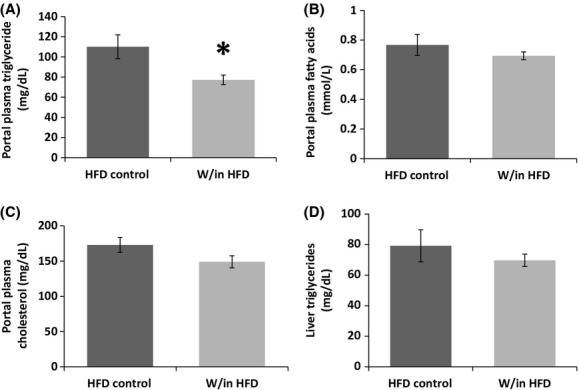
Experiment 1: autotransplantation of subcutaneous adipose tissue in HFD mice decreased portal plasma triglycerides compared with HFD controls (A). Portal plasma fatty acid and cholesterol and liver triglycerides were not alerted (B–D).

As characterized in our previous papers (Foster et al. 2010, 2011b), viable transplants are revascularized with a normal appearance both macro- and microscopically. At blunt dissection healthy transplanted adipose tissue was revascularized and contained no visible fibrosis. Mice with apparent fibrosis were not included in the study (*n* = 2). Microscopic evaluation of transplants that appeared healthy during blunt dissection confirmed normal-appearing unilocular adipocytes, with intermittent macrophages and vascular cells. The mean relative areas of the transplant and control IWAT were not different (Control Inguinal 33,756 ± 3331 and Inguinal Transplant 30,520 ± 2754; Relative average pixels). Although liver triglyceride concentration was not different among the groups (Fig. 3D), portal plasma triglycerides were significantly decreased in HFD mice with autotransplantation (Fig. 3A). Portal plasma free fatty acids and cholesterol were not changed.

### Experiment 2 – Is subcutaneous adipose tissue removal harmful or helpful in obese mice?

#### Glucose tolerance test

**Figure 4:**
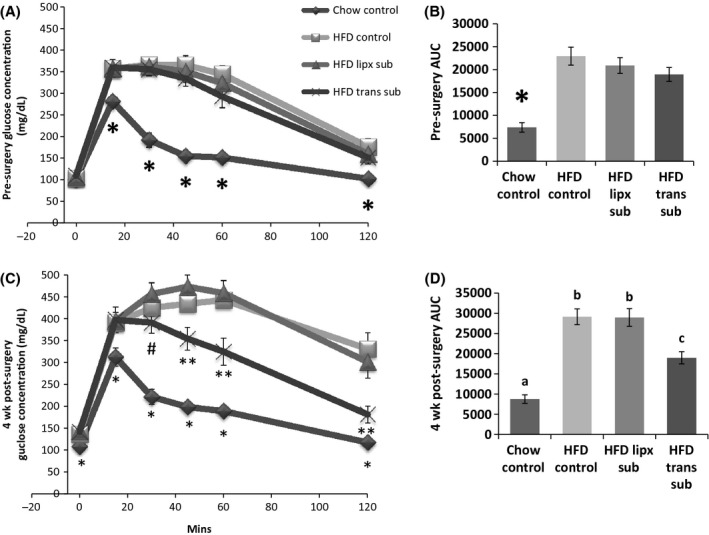
Experiment 2: glucose tolerance tests before surgery (A) and 4 weeks postsurgery (C). Before surgery HFD groups were glucose intolerant compared with chow group (*P ≤ 0.05) (A). Presurgery area under the curve was lower in chow mice (B). 4 weeks postsurgery HFD groups remained glucose intolerant compared with controls (*P ≤ 0.05 vs. control). HFD mice with heterotransplantation, however, were lower than other HFD groups 45, 60, and 120 min (**P ≤ 0.05) postinjection and also lower than subcutaneous adipose removal mice at 30 min (#*P* ≤ 0.05) (C). Area under the curve for HFD heterotransplant mice was lower than other HFD groups, but higher than chow controls (different letters indicate significance *P* ≤ 0.05) (D).

The three HFD surgery groups (Chow Control *n* = 9, HFD Control *n* = 10, HFD Trans *n* = 9, and HFD Lipx *n* = 10) were grouped as described in Experiment 1, with the addition of a chow control group for comparison purposes (Fig. 4A). HFD animals were glucose intolerant presurgery relative to chow controls as evidenced by significantly higher glucose concentrations at 15, 30, 45, 60, and 120 min postglucose injection (*P* ≤ 0.05, Fig. 4A) and an elevated AUC (*P* ≤ 0.05, Fig. 4B). Four weeks postsurgery, all HFD glucose curves, including baseline 0 mins and AUCs remained significantly higher than chow-fed controls (*P* ≤ 0.05, Fig. 4C and D). Subcutaneous adipose tissue transplantation (HFD Trans Sub), however, significantly decreased the area under the glucose curve relative to HFD control and HFD mice that underwent the subcutaneous fat removal protocol (Lipx Sub) (*P* ≤ 0.05); a result of decreased glucose concentration at 30, 60, and 120 min (*P* ≤ 0.05). In addition, HFD Trans Sub mice glucose concentration was significantly decreased compared with HFD Lipx Sub at 15 min (*P* ≤ 0.05).

#### Food intake, body weight, and tissue mass

Weekly and cumulative calories consumed were not different among HFD mice (Chow Control 395 ± 5.62 kcals, HFD Control 504 ± 15.02 kcals, HFD Lipx Sub 492.53 ± 9.03 kcals, and HFD Trans Sub 485.24 ± 11.26 kcals). All HFD mice had significantly increased visceral, epididymal, perirenal, and inguinal WAT compared with chow controls (*P* ≤ 0.05, Table 2). Consistent with this NMR fat mass and body mass were also significantly higher (*P* ≤ 0.05, Table 2). IWAT mass was significantly decreased in the HFD Lipx Sub group compared with HFD Trans Sub (*P* ≤ 0.05) because the depot was partially excised. Lean tissue mass was not different among any of the groups.

**Table 2:** Experiment 2: body mass (bilateral mass – g), NMR fat, and lean mass and absolute individual adipose tissue mass (g)

		NMR (g)					
						
	Body weight (g)	Fat	Lean	VWAT (g)	EWAT (g)	IWAT (g)	PWAT (g)
Chow control	28.7 ± 0.47*	2.180 ± 0.26*	22.74 ± 0.22	0.36 ± 0.02*	0.49 ± 0.05	0.72 ± 0.05^a^	0.22 ± 0.03*
HFD control	39.67 ± 1.62	13.13 ± 0.81	23.91 ± 0.61	0.92 ± 0.08	2.17 ± 0.13	1.78 ± 0.15^b,c^	1.10 ± 0.09
HFD Lipx Sub	38.4 ± 1.6	12.49 ± 1.06	24.1 ± 0.58	0.96 ± 0.12	2.30 ± 0.21	1.45 ± 0.16^b^	1.20 ± 0.11
HFD Trans Sub	40.22 ± 2.11	11.52 ± 1.85	25.2 ± 0.37	1.15 ± 0.18	2.20 ± 0.26	2.23 ± 0.38^c^	1.14 ± 0.15

Values are means ± SEM. NMR, nuclear magnetic resonance; VWAT, visceral white adipose tissue; EWAT, epididymal white adipose tissue; IWAT, inguinal white adipose tissue; PWAT, perirenal white adipose tissue; HFD, high-fat diet.

* *P* ≤ 0.05 control versus surgery, different letters indicate significance *P* ≤ 0.05.

#### Adipose tissue histology and portal plasma measurement

**Figure 5:**
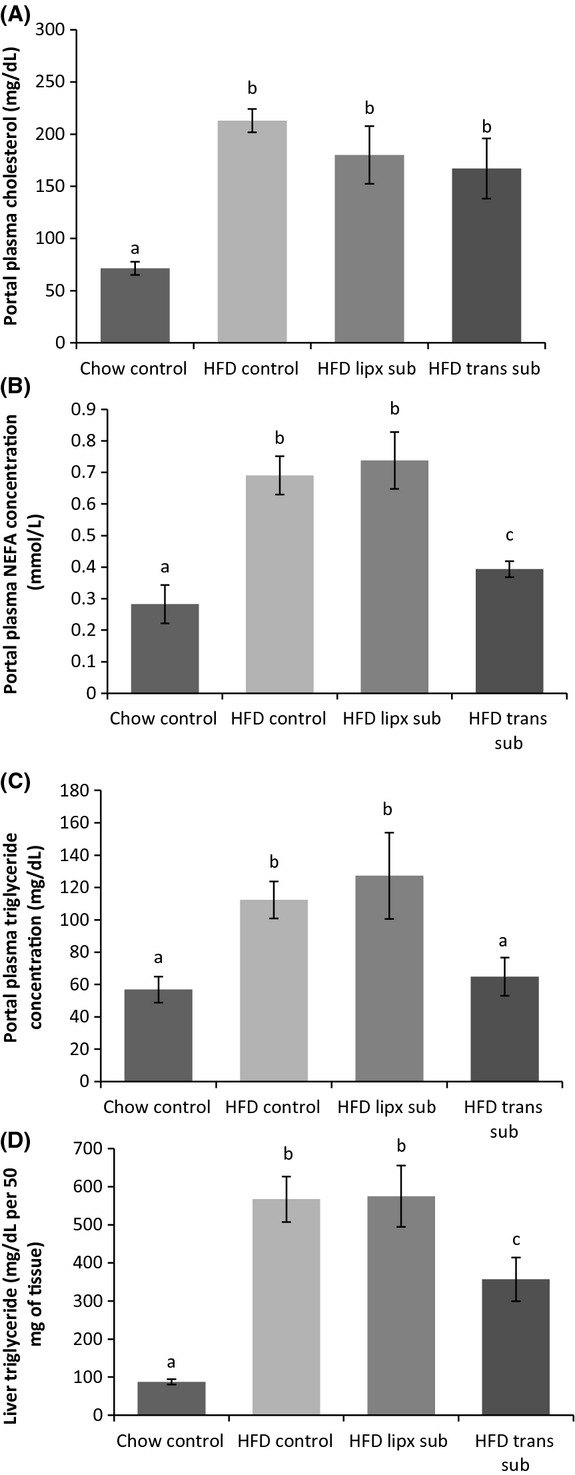
Experiment 2: HFD increased portal triglyceride (A), fatty acid (B), and cholesterol (C) concentration compared with control mice. However, HFD animals with heterotransplantation had triglyceride levels similar to chow animals (A) and lowered portal free fatty acid (B). Portal cholesterol was not altered by any surgical procedure (C). Hepatic triglyceride concentration was also increased in HFD mice, but lowered in those with heterotransplantation (D). Different letters indicate significance *P* ≤ 0.05.

**Figure 6:**
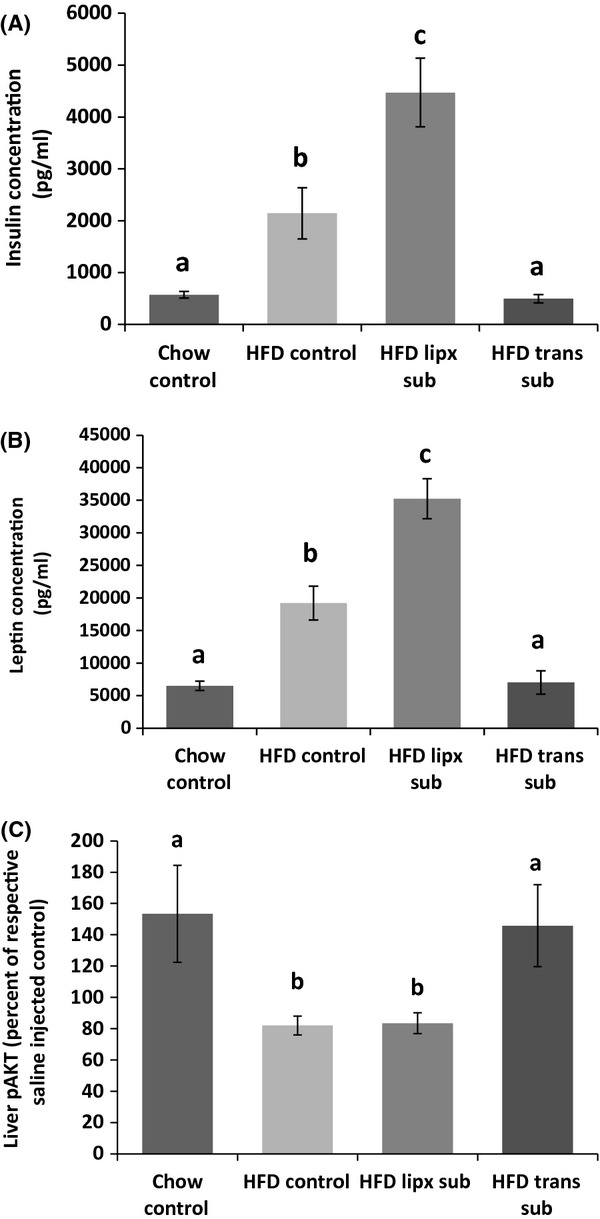
Experiment 2: leptin (A) and insulin (B) concentrations were significantly increased in all HFD groups, but those with heterotransplantations. Heterotransplantation restored insulin and leptin levels to chow control levels, whereas removal of subcutaneous adipose tissue induced increases greater than HFD control mice. Although hepatic insulin sensitivity was decreased by HFD, heterotransplantation in HFD mice restored hepatic insulin sensitivity to chow controls levels (C). Subcutaneous fat removal in HFD-fed mice did not change hepatic insulin sensitivity. Different letters indicate significance *P* ≤ 0.05.

Transplants were viable and revascularized; however, those with apparent fibrosis, were excluded from the study (*n* = 1). The mean relative area of transplanted IWAT was not different from IWAT of HFD controls (Control Inguinal 39,696 ± 6944 and Inguinal Transplant 37,391 ± 3139; Relative average pixels). Portal plasma cholesterol was significantly increased in HFD animals compared with controls (*P* ≤ 0.05; Fig. 5A); but there was no difference among HFD groups. Portal nonesterified fatty acid (NEFA) concentration was also significantly increased in all HFD groups compared with chow controls (*P* ≤ 0.05; Fig. 5B); although HFD animals with transplants were significantly lower than control and fat removed HFD groups. HFD consumption significantly increased triglyceride concentration in the portal plasma and liver compared with chow controls (*P* ≤ 0.05; Fig. 5C and D). Transplantation significantly decreased liver triglycerides compared with other HFD groups and also significantly decreased portal plasma triglycerides to levels similar to those of chow-fed control levels. Fat removal, on the other hand, did not alter lipids. Portal plasma resistin and adiponectin were not different among chow and HFD groups, but HFD significantly increased insulin and leptin concentrations (*P* ≤ 0.05; Fig. 6A and B). Insulin and leptin concentrations were further increased in animals with subcutaneous fat removed compared with HFD controls (*P* ≤ 0.05). Conversely, HFD mice with subcutaneous adipose transplants had portal plasma insulin and leptin level similar to chow control mice. Inflammatory markers MCP-1, IL-6, and PAI-1 were below detectable limits for all groups.

#### Liver insulin signaling

Liver pAKT, following insulin injection, was significantly reduced in HFD animals compared with chow control (*P* ≤ 0.05; Fig. 6C). Subcutaneous fat removal did not alter HFD-induced reductions in pAKT, but fat transplantation significantly increased pAKT compared with other HFD groups (*P* ≤ 0.05).

## Discussion

Obesity-induced metabolic dysregulation is not exclusively a consequence of increased adipose mass, but is also a consequence of altered adipose depot function (Konrad et al. 2007; Hocking et al. 2008; Tran et al. 2008; Foster et al. 2010). Hence, it is proposed that obesity-induced metabolic alterations are principally due to the quality that is intrinsic characteristics and subsequent responses to excess energy stored, rather than the quantity of adipose tissue. Our results demonstrate autotransplantation of subcutaneous adipose tissue to the visceral cavity improves glucose tolerance and decreases plasma insulin concentration and portal plasma triglycerides in HFD-induced obese mice. These alterations were independent of decreased body mass or total adiposity. Subcutaneous adipose tissue removal was also investigated independently of transplantation to identify metabolic outcomes that might arise from surgical procedures necessary for autotransplantation. In diet-induced obese mice subcutaneous adipose tissue transplantation to the visceral cavity improved glucose tolerance and hepatic insulin signaling. In addition, portal lipids, hepatic triglyceride concentration and insulin and leptin concentrations were decreased in transplant mice. None of these improvements occurred following subcutaneous adipose tissue removal; in fact insulin and leptin concentrations were elevated to levels that surpassed those of HFD control mice. Neither of the procedures altered body mass, total adiposity, or lean tissue mass. Overall we have demonstrated that transplantation via donor or homologous relocation attenuates metabolic derangement despite continued HFD feeding, whereas fat removal further enhances certain aspects.

Metabolic improvements following transplantation of subcutaneous adipose tissue to the visceral cavity have been demonstrated in lean mice maintained on standard chow (Konrad et al. 2007; Tran et al. 2008; Foster et al. 2011b) or provided a HFD coincident with surgery (Hocking et al. 2008). Consistent with this, we also demonstrated in obese glucose intolerant mice, that hetero- and autotransplantation improves glucose tolerance and decreases insulin concentration. Unlike previous studies (Hocking et al. 2008; Tran et al. 2008), however, subcutaneous transplantation did not decrease body mass and/or total adiposity. Inconsistencies between studies are likely due to study duration (5 weeks postsurgery vs. 8 or 12), and/or adiposity levels at transplantation (obese vs. lean mice) (Hocking et al. 2008; Tran et al. 2008). Longer postsurgical time in our model may have produced decreases in total adiposity given that autotransplantation decreased epididymal adipose tissue mass. The mechanisms that cause decreases in adiposity are unknown. Discrepancies in body mass, adiposity, and subsequent liver triglyceride concentration among Experiments 1 and 2 are prospectively a result of housing conditions before experiment start. Mice in Experiment 1 were grouped housed for 6 weeks before surgery where they endured constant social conflict. Because of this animals in Experiment 2 were individually housed immediately upon arrival.

Metabolic improvements following transplantation of subcutaneous adipose tissue are proposed to be due to intrinsic characteristics of the depot. Indeed, gene expression and protein secretion differ significantly among white adipose tissue depots. For example, in rodents, leptin, resistin, CD68, and MCP-1 gene expression is highest in the subcutaneous depot whereas TNFα is highest in the visceral depot (Romero Mdel et al. 2009). Moreover, secretory capacity appears to be higher in visceral compared to subcutaneous adipose tissue (Hocking et al. 2010). Although we did not investigate gene expression or protein release from adipose tissue depots, we did measure adipo/cytokine concentration in the portal blood supply following adipose tissue manipulations. Heterologous transplantation of subcutaneous adipose tissue to the visceral cavity reduced leptin concentration, which is consistent with previous systemic plasma measurements (Tran et al. 2008). Contrary to previous transplantation studies in chow-fed mice (Tran et al. 2008) leptin decreased independently of alterations in body mass in HFD mice. Although leptin is an adiposity signal many factors can alter its production and secretion regardless of adipose tissue mass such as food intake, fasting, cold exposure, and insulin (Szkudelski 2007). Insulin (Barr et al. 1997; Cheng et al. 2000) and glucose (Mizuno et al. 1996; Mueller et al. 1998; Levy and Stevens 2001) have a stimulatory effect on leptin production and secretion, therefore inhibited leptin release in this HFD transplantation model is likely a secondary consequence of decreased circulating insulin and glucose. Unlike previous studies, adiponectin neither was altered (Tran et al. 2008) nor was resistin. Although some proinflammatory cytokines were below detectable limits, others have determined it is unlikely that these cytokines, IL6 and tumor necrosis factor α (TNFα), mediate transplant-induced improvements (Tran et al. 2008).

We previously hypothesized that transplant-induced improvements were due to enhanced fatty acid uptake of the subcutaneous adipose tissue transplanted in proximity to the portal vein, thus reducing fatty acid flux into and triacylglycerol storage in the liver (Foster et al. 2011b). Here, we have demonstrated, in an obese model, that transplantation reduced portal triglyceride and free fatty acid concentration and decreased liver triglyceride stores. However, adipose tissue mass was not increased. This observation may be a consequence of the 4-week duration of the study. That is, this interval of time may be insufficient to detect significant lipid diversion to the transplant or other adipose depots. Therefore, the assessment of gene expression for lipogenic, adipogenic, and angiogenic factors may provide more insight, in this regard. There are, however, numerous factors that may decrease circulating portal lipids. For example, the enhanced hepatic insulin sensitivity following subcutaneous transplantation likely enhances the ability of insulin to decrease cholesterol and triacylglycerol secretion from the liver. Thus, markers for hepatic lipid release should be measured in the future. β-oxidation of fatty acid may also play a role in decreasing circulating lipids. Although we previously demonstrated that hepatic β-oxidation was significantly decreased following subcutaneous adipose tissue transplantation in lean rodents (Foster et al. 2011b), other tissues such as muscle or brown adipose may also play a role in mediating insulin sensitivity via alterations in β-oxidation.

In rodents, removal of adipose tissue also improves glucose tolerance and insulin sensitivity and reduces adipokine levels (Barzilai et al. 1999; Gabriely et al. 2002; Pitombo et al. 2006), however, these alterations are specific to visceral or intraabdominal adipose excision. Although subcutaneous adipose tissue is proposed to serve as a “metabolic sink,” which plays a role in obesity-induced pathophysiology of the liver, studies investigating the significance of subcutaneous adipose accumulation during energy excess are limited. Here, we have demonstrated that removal of subcutaneous adipose tissue can be detrimental because obesity-induced increases in portal insulin and leptin concentrations following removal are exacerbated in mice that underwent this procedure. Hence, removal of subcutaneous adipose tissue may trigger mechanisms that enhance insulin production and release which subsequently increase leptin release from other adipose depots. Studies have demonstrated subcutaneous adipose tissue removal increases triglyceride concentration and hepatic lipid deposition in hamsters (Weber et al. 2000), whereas others demonstrate no deleterious consequences in mice (Shi et al. 2007). Inconsistencies between studies are likely a result of differences in study duration, diet composition, and/or amount of subcutaneous adipose tissue removed. Other investigations that demonstrate subcutaneous adipose tissue removal induced metabolic deterioration are hampered by animal model (chow-fed immune-deficient mice) (Ishikawa et al. 2006) or design (acute/immediate effects of fat removal via ultrasound in chow-fed animals) (Goncalves et al. 2009).

Using hetero- and autotransplantation, we have demonstrated that the intrinsic characteristics of subcutaneous adipose tissue differ from those of visceral. Subcutaneous adipose tissue relocation to the visceral cavity improves glucose tolerance in diet-induced obese mice and decreases circulating lipids via factors that are yet to be identified. The appearance and fate of circulating and stored lipids should be further investigated, given that portal plasma lipids decrease, but adipose tissue storage was not altered. Contrarily, removal of subcutaneous adipose tissue may be detrimental in an obese model. The next step is to identify the intrinsic characteristics of visceral and subcutaneous adipose depots that drive these differential metabolic outcomes.

## Perspectives and Significance

The number of overweight/obese adults and children is steadily increasing as is the frequency of associated health consequences such as type 2 diabetes, cardiovascular disease (CVD), and high circulating lipids (cholesterol and triglycerides) (for review see Hedley et al. 2004). Consequently, obesity is a strong predictor of mortality and obesity-associated comorbidities are among the top causes of deaths in the United States. However, not all obese individuals experience secondary health consequences typically associated with body fat accumulation. This has led to the hypothesis that the distribution of fat in the body is an important determinant of adverse health outcomes. It is established that central or intraabdominal obesity (the “apple” shape) is a risk factor for many adverse metabolic outcomes such as hypertension (Cassano et al. 1990; Seidell et al. 1991), type 2 diabetes (Chan et al. 1994; Carey et al. 1997; Astrup et al. 2007), and CVD (Sowers 2003), whereas peripheral or subcutaneous obesity (the “pear” shape) is associated with a reduced risk for hypertension (Rimm et al. 1988), type 2 diabetes (Snijder et al. 2004), and CVD (Porter et al. 2009; Manolopoulos et al. 2010). It has been proposed that the “apple” shape is deleterious due, in part, to excess fatty acid flux to the liver via the hepatic portal vein whereas the “pear” shape provides protection by serving as a long-term energy storage unit that collects lipids that might otherwise lead to problems in nonadipose tissues (e.g., heart, liver, pancreas). Although it is recognized that adipose tissue dysfunction plays a role in obesity-induced metabolic dysregulation relatively little is known about mechanisms that control the protective effects of the “pear” adipose tissue distribution or the negative effects of the “apple.” The present experiments demonstrate via adipose tissue manipulation in a diet-induced obese animal model the role of central (visceral) and peripheral (subcutaneous) adipose tissue in metabolic regulation. The procedure of adipose tissue removal with or without subsequent transplantation validates that increased fat mass is not the fundamental problem in obesity-related metabolic disorders, rather adipose tissue quality is. The addition of subcutaneous adipose tissue to the visceral cavity mimicking visceral fat accumulation improved metabolic indices in obese mice because of its differential inherent characteristics. Conversely, removal (fat loss) of subcutaneous adipose tissues under the same circumstances exacerbated aspects of metabolic dysregulation. This data supports the “metabolic sink” postulate which proposes the protective effects of peripheral adipose tissue occur via its ability to sequester surplus lipid (Kral 1975, 1988). Overall, the identification of mechanisms/factors that make subcutaneous adipose tissue beneficial may help develop therapeutic treatments for prevention or reversal of obesity comorbidities.
